# Supplementary material for: Lack of elevated pre-ART elastase-ANCA levels in patients developing TB-IRIS
Source: PLoS One. 2020 Dec 31;15(12):e0244800. doi: 10.1371/journal.pone.0244800 (PMC7775098; doi:10.1371/journal.pone.0244800)
Supplement: Attachment — Submitted filename: Manuscript, ANCA levels in TB-IRIS v1.3_TC.doc [file pone.0244800.s002.doc]

**Lower pre-ART levels of elastase-ANCA in patients developing TB-IRIS**

ANCA levels in TB-IRIS

Odin Goovaerts^1^, Marguerite Massinga-Loembé^1,2,3^, Pascale Ondoa^4,9^, Ann Ceulemans^1^, William Worodria^5,6,7^, Harriet Mayanja-Kizza^5,6^, Robert Colebunders^8^, Luc Kestens^1^, the TB-IRIS Study Group^¶^.

^1^ Department of Biomedical Sciences, Institute of Tropical Medicine, Antwerp, Belgium

^2^ Centre de Recherches Médicales de Lambaréné (CERMEL), Albert Schweitzer Hospital, Lambarene, Gabon

^3^ Institut für Tropenmedizin,Universität Tübingen, Tübingen, Germany

^4^ African Society for Laboratory Medicine, Addis Ababa, Ethiopia

^5^ Department of Medicine, Mulago Hospital, Kampala, Uganda

^6^ Infectious Diseases Institute, Makerere University College of Health Sciences, Kampala, Uganda

^7^ Infectious Diseases Network for Treatment and Research in Africa (INTERACT), Kampala, Uganda

^8^ Global Health Institute, University of Antwerp

^9^ Amsterdam Institute of Global Health and Development, Academic Medical Center, Department of Global Health, University of Amsterdam, The Netherlands

* Corresponding author

ogoovaerts@itg.be (OG)

^¶^The complete membership of the author group can be found in the Acknowledgements.

## Abstract

**Background**

Tuberculosis-associated immune reconstitution inflammatory syndrome (TB-IRIS) in HIV-TB co-infected patients receiving antiretroviral therapy (ART) has been linked to neutrophil activation. Anti-neutrophil cytoplasmic antibodies (ANCAs) are also associated with neutrophil activation. Since ANCAs are reportedly skewed in TB and HIV infections, we investigated plasma levels of 7 ANCAs in TB-IRIS patients.

**Methods**

We retrospectively compared 17 HIV-TB patients who developed TB-IRIS with controls of similar CD4 count, age and gender who did not (HIV^+^TB^+^ n=17), HIV-patients without TB (HIV^+^TB^-^, n=17) and 10 HIV-negative (HIV^-^TB^-^) controls. Frozen plasma was collected before ART, at 3 and 9 months of ART, and examined by ELISA for levels of 7 ANCAs directed against; Proteinase 3 (PR3), Myeloperoxidase (MPO), Permeability-increasing protein (BPI), Elastase, Cathepsin, Lysozyme, and Lactoferin.

**Results**

Compared to HIV^+^TB^+^ controls, pre-ART anti-elastase levels were lower in TB-IRIS patients (p = 0.026) and HIV^-^TB^-^ controls (p = 0.044), whereas other ANCAs did not show significant differences between groups at any time point. A significant decrease over time could be observed in TB-IRIS patients during ART for anti -PR3 (p = 0.027), -lysozyme (p = 0.011), and -lactoferin (p = 0.019). Conversely, HIV^+^TB^+^ controls showed a significant decrease over time for anti -MPO (p = 0.002), -lyzosyme (p = 0.002) and -elastase (p < 0.001).

**Conclusion**

Lower pre-ART anti-elastase levels in TB-IRIS patients correspond to previous findings of lowered immune capacity in patients that will develop TB-IRIS. This may suggest a specific role for anti-elastase, elastase or even matrix-metalloproteinases in TB-IRIS. The precise dynamics of neutrophil activation in HIV-TB merits further investigated and could provide more insight in the early mechanisms leading up to TB-IRIS.

## Introduction

A subgroup of HIV patients with a tuberculosis (TB) co-infection who start successful antiretroviral therapy (ART) are at risk of developing a complication called paradoxical TB-associated immune reconstitution inflammatory syndrome (TB-IRIS), despite responding well to preceding TB-treatment [1,2]. TB-IRIS typically develops within the first 2 months after starting ART, with the majority of cases occurring before 1 month when CD4+ T-cells are being replenished [3,4]. Though underlying tissue destructive inflammation is heavily associated with TB-IRIS, the immune-pathogenesis is still not well understood [5].Patients often require additional therapy and/or hospitalization, presenting with a wide array of symptoms. This heterogeneity provides a challenge to distinguish TB-IRIS from other complication, since diagnosis of TB-IRIS still mainly relies on clinical examinations. Though a decade of research is steadily uncovering the mechanisms behind TB-IRIS, there is still a need for reliable laboratory markers to accurately predict patients at risk of the syndrome [6].

Early studies described a high TB-antigen burden, a short interval between TB treatment and ART, and a low CD4+ T cell count prior to ART initiation as the major risk factors of TB-IRIS [7–9]. However, not all HIV-TB patients with advanced disease develop TB-IRIS. Therefore, additional mechanisms such as an atypical restoration of immune responses to TB, or an uncoupling of adaptive and innate immune responses, have been proposed as underlying processes leading up to TB-IRIS [3,10–12]. Whereas an early role of innate immune cells in the inflammatory cascade during TB-IRIS has become increasingly apparent [13–18], it still remains unclear which pre-ART factors prime the immune system for the inflammatory burst during ART. With that in mind, current theories seem to flow together to form a more complex model of TB-IRIS pathogenesis that spans different aspects of the immune system [19].

Anomalies in the innate arm of the immune system have been previously reported by different studies. The IRIS-typical cytokine storm seems to be dominated by innate factors such as IL-6, which suggests the involvement of monocytes [14,20–24]. We previously observed lower levels of IL-6 and LPS-binding protein in TB-IRIS patients prior to starting ART, suggesting less innate immune activation during the TB-treatment – ART interval [14]. Conversely, neutrophils and related products such as matrix-metalloproteinases have been closely associated with the tissue-damage observed during ART in TB-IRIS patients [25,26]. In fact, plasma concentrations of human neutrophil peptide 1-3 and elastase were found to be elevated during TB-IRIS events [27]. Therefore, investigating downstream factors involved in monocyte/neutrophil activation and tissue damage could provide additional markers, which could help elucidate the pathogenesis of TB-IRIS.

Taking a page out of the book of auto-immune diseases, anti-neutrophil cytoplasmic antibodies (ANCAs, a group of antibodies directed against different neutrophilic components), have been widely linked to neutrophil activation and tissue damage in small vessel vasculitides [28,29]. While anti-Myeloperoxidase (MPO) and anti-Proteinase 3 (PR3) are considered the major components of the ANCA group, antibodies against other components such as bacterial permeability-increasing protein (BPI), elastase, cathepsin, lysozyme, and lactoferin have all been described in inflammatory conditions which include; inflammatory rheumatic diseases, inflammatory bowel disease, etc. [30–33]. Interestingly, aberrant ANCA plasma levels have also been associated with chronic infections such as HIV and TB, perhaps triggered by molecular mimicry or persistent host responses to the infection [34–36]. Nonetheless, ANCA findings in HIV and TB remain inconsistent. The objective in this study was therefore to explore ANCA levels in TB-IRIS patients and controls. We hypothesized that pre-ART ANCA levels would be lower in TB-IRIS patients, thus reflecting diminished pathogen clearing in these patients. Nested as a case-control study within a large prospective cohort of TB-IRIS patients, we describe lower pre-ART plasma levels of anti-Elastase, but not other ANCAs, in TB-IRIS patients compared to HIV-TB controls.

## Methods

**Study population**

Patients in our study were recruited as part of a prospective observational study on the clinical spectrum of HIV-TB and TB-IRIS between 2007 and 2012 at Mulago Hospital, Kampala, Uganda [7,37,38]. Enrollment included a cohort of HIV-TB co-infected adults (HIV^+^TB^+^) who had been treated for active TB infection for less than 2 months, and a cohort of HIV-patients without clinical signs of TB co-infection (HIV^+^TB^-^). Also, a group of HIV-negative controls without active TB (HIV^-^TB^-^) was recruited. Patients were excluded in case of prior use of ART, pregnancy, or Grade 3 liver or renal abnormalities. According to Ugandan national guidelines, HIV-patients received a non-nucleoside reverse transcriptase inhibitor-based ART. The median interval between starting TB-treatment and ART was 6 weeks for the whole cohort, including adherence preparation for HIV^+^TB^+^ patients. HIV-patients were followed up for 10 months in order to monitor paradoxical TB-IRIS development. Sixty (24%) out of 254 HIV-TB co-infected patients developed IRIS (TB-IRIS), whereas HIV^+^TB^+^ patients who did not develop IRIS-related symptoms served as non-IRIS controls. In this study, a subset of patients with available plasma samples were selected from each patient-group. HIV-patients had blood samples taken before initiation of ART, at 3 months and at 9 months after starting ART, while HIV-negative controls received no ART and thus had samples taken only once.

**Definitions**

*Mycobacterium tuberculosis* infection was diagnosed according to the TB/HIV WHO guidelines [39]. Clinical tests to confirm TB infection included: clinical examination, chest X-rays, abdominal ultrasounds, sputum smear microscopy for acid-fast bacilli, and mycobacterial culture of sputum, aspirate or effusion if available. All suspected TB-IRIS cases were evaluated by the study physicians according to the International Network for the Study of HIV-associated IRIS (INSHI) clinical case-definition, and subsequently classified as TB-IRIS cases by a committee of two co-authors (RC and WW) [1]. TB-IRIS was diagnosed when patients presented with at least 1 major criterion (e.g. enlarged lymph nodes) or 2 minor criteria (e.g. fever and cough) and treatment failure was excluded by monitoring viral loads and CD4 counts.

**Plasma ANCA analysis**

Venous blood was drawn into EDTA tubes and plasma was separated from cells by centrifugation and stored at -80°C. Plasma samples were subsequently thawed and diluted 1:101. Plasma concentrations of 7 ANCA’s (PR3, MPO, BPI, Elastase, Cathepsin, Lysozyme, Lactoferin) were determined by ELISA according to the manufacturer’s instructions (AESKU.DIAGNOSTICS GmbH & Co., Wendelsheim, Germany).

**Ethics Statement**

The study was approved by: the Research Committee of the Infectious Diseases Institute (IDI), the ethical review board of Makerere University (IRB-Makerere-05_2007), the Uganda National Council of Science and Technology, the institutional review board of the Institute of Tropical Medicine of Antwerp and the Ethics Committees of the Faculties of Medicine of the University of Antwerp (CME_UZA_7/29/157). Written informed consent was obtained from all study participants.

**Statistical analysis**

## SPSS software (version 17.0) and GraphPad Prism (version 7) was used for statistical analysis, with significance level set at p < 0.05. Kruskal-Wallis tests or Mann-Whitney U and Fisher’s exact tests when comparing >3 groups were used to analyse differences between patient groups. Changes over time were analysed for each group using Friedman tests (n = 11 in each group, since complete follow up samples are needed for longitudinal analysis). Dunn’s multiple comparison post-hoc tests and multiplicity adjusted p-values were used to indicate differences between specific groups and time points.

## Results

**Study population**

Nested within a prospective cohort study for TB-IRIS, we compared 17 TB-IRIS patients to equal numbers of HIV^+^TB^+^ and HIV^+^TB^-^ controls. The median (interquartile range (IQR)) number of days between starting ART and TB-IRIS diagnosis was 14 (11-27) days. None of the HIV-infected groups showed significant differences in clinical pre-ART characteristics, except for HIV^+^TB^-^ controls who showed lower CRP levels compared to TB-IRIS patients and HIV^+^TB^+^ controls (p <0.001, Table 1). TB-IRIS patients with available HIV viral loads (n=11) showed a significant decline in viral load after 2 weeks of ART [5,4 (5,2 - 5,7) vs. 3,4 (3,2 - 3,4) Log copies/ml, p = 0.001 using Wilcoxon signed-rank test]. In addition, 10 HIV^-^TB^-^ controls were selected. HIV^-^TB^-^ controls did not differ in age or sex from any of the HIV-infected groups, but had lower CRP levels [0.9 (0.55-1.60) mg/L] compared to TB-IRIS patients and HIV^+^TB^+^ controls (p ≤ 0.001).

**Table 1. Clinical characteristics of the study population.**

|  | **TB-IRIS**  _(n=17)_ | **HIV^+^TB^+^**  _(n=17)_ | **HIV^+^TB^-^**  _(n=17)_ | **p^a^** |
| --- | --- | --- | --- | --- |
| Male (n) (%) | 8/17 (47) | 8/17 (47) | 8/17 (47) | 1.000^b^ |
| Age (Years) | 29 (25 - 39) | 30 (25 - 37) | 35 (30 - 38) | 0.412 |
| CRP (mg/L) | 7,6 (5,8 – 42,0)^e^ | 13,0 (4,8 – 39,0) | 1,3 (0,7 - 4,4) | <0.001^c^ |
| # CD4 (cells/µl) | 38 (9,5 - 134) | 32 (14 - 123) | 34 (13 - 133) | 0.974 |
| Treatment interval^d^ | 44 (26 - 70) | 58 (36 - 65)^f^ | - | 0.634^h^ |
| Temperature (°C) | 36 (36 - 37) | 36 (36 - 37) | 36 (36 - 37)^g^ | 0.435 |

Data are represented as median and interquartile range unless stated otherwise. ^a^Kruskal-Wallis tests were used to compare groups unless stated otherwise. ^b^Fisher’s exact test ^c^Differences between specific groups were calculated using Dunn’s post-hoc test with multiplicity adjusted p-values: TB-IRIS vs. HIV^+^TB^+^ p>0.999; TB-IRIS vs. HIV^+^TB**^-^ p=0.005**; HIV^+^TB^+^ vs. HIV^+^TB**^-^ p=0.009**. ^d^#days between initiation of TB-treatment and ART. ^e^n=13. ^f^n=15. ^g^n=15.^h^Mann-Whitney U test. The level of significance was set to P > 0.05 for all tests. CRP = C-Reactive Protein.

**Anca levels in TB-IRIS patients and controls**

Given the association of ANCAs with inflammatory conditions, we explored plasma levels of 7 different ANCAs in TB-IRIS patients and controls at different intervals during treatment (Fig 1A). Prior to ART, we observed significantly lower levels of anti-elastase in TB-IRIS patients (p = 0.026) and HIV^-^TB^-^ controls (p = 0.044) compared to HIV^+^TB^+^ controls. Though anti-elastase levels remained low in TB-IRIS patients, this significance was lost at 3 months and 9 months of ART, since HIV^+^TB^+^ controls showed a significant decrease in anti-elastase levels over time (p < 0.001; Fig 1B). Dunn’s post-hoc test revealed a decrease from month 3 to month 9 (p = 0.009) and pre-ART to month 9 (p = 0.002).

**Fig 1. Plasma levels of anti-elastase in TB-IRIS patients and controls.** Graphs show a comparison between groups (A), as well as changes over time for individual patients (B) in the plasma levels of anti-elastase (U/ml) measured prior to ART, and at 3 months and 9 months of ART. Significant overall differences between TB-IRIS patients (red circles), HIV^+^TB^+^ (blue triangles), HIV^+^TB^-^ (green squares), and HIV^-^TB^-^ (yellow hexagons) controls were calculated using Kruskal-Wallis tests, with Dunn’s post-hoc test shown as capped lines to highlight specific differences between individual groups (A). Significant variation over time was calculated for each group using Friedman tests, with Dunn’s post-hoc test shown as capped lines to highlight specific differences between individual time points (B). Out of 17 TB-IRIS patients with samples available pre-ART, only 11 had samples available at both month 3 and month 9. The total number of patients analysed over time was thus 11 for each group. Analysis were performed between all groups at each time point, and between every time points for each group with the level of significance was set to P < 0.05. Non-significant p-values have been omitted from the graphs. Overall Kruskal-Wallis p-value was ^a^p = 0.009 and overall Friedman p-value was ^b^p ≤ 0.001.

In contrast, no significant differences could be observed between groups at any time point for anti- PR3, -MPO, -BPI, -elastase, -cathepsin, -lysozyme, and -lactoferin (Fig 2). However, a significant decrease over time could be observed in TB-IRIS patients for anti -PR3 (p = 0.027), -lysozyme (p = 0.011), and -lactoferin (p = 0.019; Fig 3A,E,F). Dunn’s post-hoc test showed a main decrease between pre-ART and the month 9 time point (p = 0.032, p = 0.017, and p = 0.017, respectively). Conversely, HIV^+^TB^+^ controls showed a significant decrease over time for anti-MPO and anti-lyzosyme (p = 0.002 for both; Fig 3B,E). For anti-MPO, this decrease was located between month 3 and month 9 (p = 0.032), as well as between pre-ART and month 9 (p = 0.004). For anti-lyzozyme, the decrease was located between the pre-ART and month 9 time point (p= 0.004).

**Fig 2. Plasma levels of other ANCAs in TB-IRIS patients and controls.** Graphs show a comparison between TB-IRIS patients (red circles), HIV^+^TB^+^ (blue triangles), HIV^+^TB^-^ (green squares), and HIV^-^TB^-^ (yellow hexagons) in the plasma levels of anti- PR3 (A), -MPO (B), -BPI (C), -cathepsin (D), -lysozyme (E), and -lactoferin (F) (U/ml) measured prior to ART, and at 3 months and 9 months of ART. Analysis were performed between all groups at each time point, with the level of significance set to P < 0.05. Non-significant p-values have been omitted from the graphs, as none were observed.

**Fig 3. Plasma levels of other ANCAs over time in TB-IRIS patients and controls.** Graphs show changes over time for individual patients in the plasma levels of anti- PR3 (A), -MPO (B), -BPI (C), -cathepsin (D), -lysozyme (E), and -lactoferin (F) (U/ml) measured prior to ART, and at 3 months and 9 months of ART. Significant variation over time in TB-IRIS patients (red circles), HIV^+^TB^+^ (blue triangles), and HIV^+^TB^-^ (green squares) controls was calculated for each group using Friedman tests, with Dunn’s post-hoc test shown as capped lines to highlight specific differences between individual time points. Out of 17 TB-IRIS patients with samples available pre-ART, only 11 had samples available at both month 3 and month 9. The total number of patients analysed over time was thus 11 for each group. No variation over time was calculated for HIV^-^TB^-^ controls, since only 1 time point was available. Analysis were performed between all groups at each time point, and between every time points for each group with the level of significance was set to P < 0.05. Overall Friedman p-values were ^a^p = 0.027, ^b^p = 0.002, ^c^p = 0.011, ^d^p = 0.002, ^e^p = 0.019.

## Discussion

TB-IRIS studies across the years have steadily been revealing an important role of the innate immune system in the inflammatory cascade that characterizes the disease [14,15,17,18]. In fact, aberrant monocyte and neutrophil reactions have been implicated in the tissue damage observed during the ongoing inflammatory process that underlies TB-IRIS [27,40]. However, despite progress being made to uncover the mechanisms behind TB-IRIS, it still remains unclear which pre-ART factors predispose the immune-system to trigger this excessive inflammatory response upon ART initiation. Studies on vasculitis have shown that neutrophils express auto-antigens when exposed to antigens [28]. Subsequent binding of so called anti-neutrophil cytoplasmatic antibodies, or ANCAs, leads to the activation of neutrophils and the release of reactive oxygen species, inflammatory cytokines and the corresponding tissue damage. Taking into account the possible roles of ANCAs in infectious diseases such as TB and HIV, as well as the apparent role of neutrophils in TB-IRIS, we aimed to explore plasma-levels of 7 different ANCAs in TB-IRIS patients prior to ART, and after 3 and 9 months of follow-up. Since we previously observed lower pre-ART levels of IL-6 and acute phase protein (lipopolysaccharide-binding protein) in TB-IRIS patients, we hypothesized that ANCA levels would also be lower in TB-IRIS patients, thus reflecting diminished pathogen clearing in these patients.

We thus investigated plasma levels of anti-PR3, -MPO, -BPI, -elastase, -cathepsin, -lysozyme, and -lactoferin in TB-IRIS patients and controls prior to starting ART. Samples taken during 3 and 9 months of ART were also analysed in order to better understand the dynamics of these ANCA’s during treatment. Contrary to our hypothesis, most ANCAs that were measured did not show significant direct differences between groups. However, anti-elastase did show lower pre-ART levels in TB-IRIS patients, in line with our theory. In addition, while both TB-IRIS patients and HIV^+^TB^+^ controls showed decreasing levels of anti-lysozyme during follow-up, TB-IRIS patients showed a stronger decrease in anti-lactoferin, whereas HIV^+^TB^+^ controls showed a decrease in anti-MPO and -elastase. This finding could suggest diverging ANCA dynamics in TB-IRIS patients, though further research is required to fully understand the dynamics spanning the TB-IRIS event. Interestingly, an increase in elastase levels has previously been described during ongoing TB-IRIS [27], though no differences were observed prior to ART. While not completely in line with nor contradicting our findings, this does suggest a more prominent role for elastase and its corresponding ANCA in TB-IRIS development. Neutrophilic elastase is associated with tissue remodelling/damage through the activation of matrix-metalloproteinases, which in and of themselves have been linked to TB-IRIS as well [26,41]. On one hand, the lower pre-ART anti-elastase levels observed here could indicate a higher level of elastase being produced and consequently binding with these ANCAs. However, our previous observations show a downregulation of immune factors prior to ART, suggesting a lowered capacity of the immune system in TB-IRIS patients to clear the mycobacterial load before ART is initiated [14,42,43]. Since our current findings correspond to our earlier results, it seems more likely that the lower anti-elastase levels are reflective of decreased neutrophil activation and, allowing a build-up of antigens that primes the immune system to over-react when ART is initiated. Nonetheless, it remains unclear why other ANCAs did not share this pattern.

As ours was a retrospective study on a condition with unpredictable timing, we could not include plasma taken during TB-IRIS event. We therefore cannot provide any insight in the evolution of ANCA levels during the ongoing TB-IRIS inflammation. Nonetheless, our selected samples span a timeframe both before and after TB-IRIS development, allowing an overview of the events surrounding TB-IRIS development. Moreover, our main finding was observed prior to starting ART, and is therefore not influenced by not including samples taken during the TB-IRIS event.

In conclusion, our study observed lower pre-ART plasma levels of anti-elastase antibodies in TB-IRIS as compared to HIV^+^TB^+^ controls, to a level similar of HIV-TB uninfected controls. Nonetheless, none of the other ANCAs were significantly different between groups. Our findings correspond to previous findings of diminished immune-activity in TB-IRIS patients prior to starting ART, and could underscore a specific role for anti-elastase, elastase or even matrix-metalloproteinases in TB-IRIS. The precise dynamics of neutrophil activation in HIV-TB patients prior to starting ART merits further investigated and could provide more insight in the early mechanisms leading up to TB-IRIS.

## Acknowledgements

The authors thank the study participants and the study team: D. Mazakpwe, K. Luzinda, P. Lwanga, M. Nakuya, C.O Namujju, C. Ahimbisibwe, J. Namaganda, A. Andama, E. Bazze and H. Kisembo. We thank N. Pakker and the data staff of the Infectious Diseases Network for Treatment and Research in Africa (INTERACT) for assistance with data monitoring and management.

**Members of the TB-IRIS study group:** Institute of Tropical Medicine, Antwerp, Belgium: Luc Kestens, Robert Colebunders, Marguerite Massinga Loembé; Infectious Disease Institute, Kampala, Uganda: Harriet Mayanja, William Worodria; Joint Clinical Research Centre: Harriet Mayanja; Université Libre de Bruxelles, Belgium: Francoise Mascart; VIB, Brussels, Belgium and Vrije Universiteit Brussel, Brussels, Belgium: Rafael van den Bergh; Institut Pasteur de Lille, France: Camille Locht; Academic Medical Centre, Department of Global Health and Amsterdam Institute for Global Health and Development, Amsterdam, The Netherlands: Peter Reiss, Frank Cobelens, Pascale Ondoa, Nadine Pakker; INTERACT, Kampala, Uganda: Roy Mugerwa, Harriet Mayanja, Nadine Pakker, William Worodria.

## Reference List

1.	Meintjes G, Lawn SD, Scano F, Maartens G, French MA, Worodria W, et al. Tuberculosis-associated immune reconstitution inflammatory syndrome: case definitions for use in resource-limited settings. Lancet Infect.Dis. pp. 516–523.

2.	Müller M, Wandel S, Colebunders R, Attia S, Furrer H, Egger M. Immune reconstitution inflammatory syndrome in patients starting antiretroviral therapy for HIV infection: a systematic review and meta-analysis. Lancet Infect Dis. 2010;10: 251–61. doi:10.1016/S1473-3099(10)70026-8

3.	Barber DL, Andrade BB, Sereti I, Sher A. Immune reconstitution inflammatory syndrome: the trouble with immunity when you had none. Nat Rev Microbiol. Nature Publishing Group; 2012;10: 150–6. doi:10.1038/nrmicro2712

4.	Murdoch DM, Venter WDF, Van Rie A, Feldman C. Immune reconstitution inflammatory syndrome (IRIS): review of common infectious manifestations and treatment options. AIDS Res Ther. 2007;4: 9. doi:10.1186/1742-6405-4-9

5.	Burman W, Weis S, Vernon A, Khan A, Benator D, Jones B, et al. Frequency , severity and duration of immune reconstitution events in HIV-related tuberculosis SUMMARY. 2007;11: 1282–1289.

6.	Worodria W, Conesa-Botella A, Kisembo H, McAdam KP, Colebunders R. Coping with TB immune reconstitution inflammatory syndrome. Expert Rev Respir Med. 2009;3: 147–52. doi:10.1586/ers.09.8

7.	Conesa-Botella A, Loembé MM, Manabe YC, Worodria W, Mazakpwe D, Luzinda K, et al. Urinary lipoarabinomannan as predictor for the tuberculosis immune reconstitution inflammatory syndrome. J Acquir Immune Defic Syndr. 2011;58: 463–8. doi:10.1097/QAI.0b013e31823801de

8.	Worodria W, Massinga-Loembe M, Mazakpwe D, Luzinda K, Menten J, Van Leth F, et al. Incidence and predictors of mortality and the effect of tuberculosis immune reconstitution inflammatory syndrome in a cohort of TB/HIV patients commencing antiretroviral therapy. J Acquir Immune Defic Syndr. 2011;58: 32–7. doi:10.1097/QAI.0b013e3182255dc2

9.	Vignesh R, Swathirajan CR, Solomon SS, Shankar EM, Murugavel KG. Risk factors and frequency of tuberculosis-associated immune reconstitution inflammatory syndrome among HIV/Tuberculosis co-infected patients in Southern India. Indian J.Med.Microbiol. pp. 279–281.

10.	Lawn SD, Meintjes G. Pathogenesis and prevention of immune reconstitution disease during antiretroviral therapy. Expert Rev Anti Infect Ther. 2011;9: 415–30. doi:10.1586/eri.11.21

11.	Grant PM, Komarow L, Lederman MM, Pahwa S, Zolopa AR, Andersen J, et al. Elevated interleukin 8 and T-helper 1 and T-helper 17 cytokine levels prior to antiretroviral therapy in participants who developed immune reconstitution inflammatory syndrome during ACTG A5164. J Infect Dis. 2012;206: 1715–23. doi:10.1093/infdis/jis604

12.	Goovaerts O, Massinga-Loembé M, Ondoa P, Ceulemans A, Worodria W, Mayanja-Kizza H, et al. Increased KLRG1 and PD-1 expression on CD8 T lymphocytes in TB-IRIS. PLoS One. 2019;14: 1–15. doi:10.1371/journal.pone.0215991

13.	Ruhwald M, Ravn P. Immune reconstitution syndrome in tuberculosis and HIV-co-infected patients: Th1 explosion or cytokine storm? AIDS. pp. 882–884.

14.	Goovaerts O, Jennes W, Massinga-Loembe M, Ceulemans A, Worodria W, Mayanja-Kizza H, et al. LPS-binding protein and IL-6 mark paradoxical tuberculosis immune reconstitution inflammatory syndrome in HIV patients. PLoS.One. 2013. pp. e81856-.

15.	Meintjes G, Skolimowska KH, Wilkinson K a, Matthews K, Tadokera R, Conesa-Botella A, et al. Corticosteroid-modulated immune activation in the tuberculosis immune reconstitution inflammatory syndrome. Am J Respir Crit Care Med. 2012;186: 369–77. doi:10.1164/rccm.201201-0094OC

16.	Ravimohan S, Tamuhla N, Steenhoff AP, Letlhogile R, Nfanyana K, Bellamy SL, et al. Immunological profiling of tuberculosis-associated immune reconstitution inflammatory syndrome and non-immune reconstitution inflammatory syndrome death in HIV-infected adults with pulmonary tuberculosis starting antiretroviral therapy: a prospective obse. Lancet Infect.Dis. pp. 429–438.

17.	Stone SF, Price P, Keane NM, Murray RJ, French M a. Levels of IL-6 and soluble IL-6 receptor are increased in HIV patients with a history of immune restoration disease after HAART. HIV Med. 2002;3: 21–7. Available: http://www.ncbi.nlm.nih.gov/pubmed/12059947

18.	Tadokera R, Meintjes G, Skolimowska KH, Wilkinson KA, Matthews K, Seldon R, et al. Hypercytokinaemia accompanies HIV-tuberculosis immune reconstitution inflammatory syndrome. 2011;37: 1248–1259. doi:10.1183/09031936.00091010.Hypercytokinaemia

19.	Cevaal PM, Bekker LG, Hermans S. TB-IRIS pathogenesis and new strategies for intervention: Insights from related inflammatory disorders. Tuberculosis. Elsevier; 2019;118: 101863. doi:10.1016/j.tube.2019.101863

20.	Price P, Mathiot N, Krueger R, Stone S, Keane NM, French MA. Immune dysfunction and immune restoration disease in HIV patients given highly active antiretroviral therapy. J.Clin.Virol. pp. 279–287.

21.	Lai RPJ, Meintjes G, Wilkinson KA, Graham CM, Marais S, Van Der Plas H, et al. HIV-Tuberculosis-Associated immune reconstitution inflammatory syndrome is characterized by Toll-like receptor and inflammasome signalling. Nat Commun. 2015;6. doi:10.1038/ncomms9451

22.	Wiesner DL, Boulware DR. Cryptococcus-Related Immune Reconstitution Inflammatory Syndrome(IRIS): Pathogenesis and Its Clinical Implications. Curr.Fungal.Infect.Rep. pp. 252–261.

23.	Andrade BB, Singh A, Narendran G, Schechter ME, Nayak K, Subramanian S, et al. Mycobacterial antigen driven activation of CD14++CD16- monocytes is a predictor of tuberculosis-associated immune reconstitution inflammatory syndrome. PLoS Pathog. 2014;10: e1004433. doi:10.1371/journal.ppat.1004433

24.	Tan DB a, Lim A, Yong YK, Ponnampalavanar S, Omar S, Kamarulzaman A, et al. TLR2-induced cytokine responses may characterize HIV-infected patients experiencing mycobacterial immune restoration disease. AIDS. 2011;25: 1455–60. doi:10.1097/QAD.0b013e328348fb18

25.	Ravimohan S, Tamuhla N, Kung SJ, Nfanyana K, Steenhoff AP, Gross R, et al. Matrix Metalloproteinases in Tuberculosis-Immune Reconstitution Inflammatory Syndrome and Impaired Lung Function Among Advanced HIV/TB Co-infected Patients Initiating Antiretroviral Therapy. EBioMedicine. pp. 100–107.

26.	Tadokera R, Meintjes GA, Wilkinson KA, Skolimowska KH, Walker N, Friedland JS, et al. Matrix metalloproteinases and tissue damage in HIV-tuberculosis immune reconstitution inflammatory syndrome. Eur.J.Immunol. pp. 127–136.

27.	Nakiwala JK, Walker NF, Diedrich CR, Worodria W, Meintjes G, Wilkinson RJ, et al. Neutrophil Activation and Enhanced Release of Granule Products in HIV-TB Immune Reconstitution Inflammatory Syndrome. J Acquir Immune Defic Syndr. 2018;77: 221–229. doi:10.1097/QAI.0000000000001582

28.	Fijolek J, Wiatr E, Petroniec V, Augustynowicz-Kopec E, Bednarek M, Gawryluk D, et al. Antineutrophil cytoplasmic antibodies and their relationship with disease activity and presence of staphylococcal superantigens in nasal swabs in patients having granulomatosis with polyangiitis: results of a study involving 115 patients from a single cen. Clin Rheumatol. Clinical Rheumatology; 2019;38: 3297–3305. doi:10.1007/s10067-019-04693-0

29.	Hu N, Westra J, Kallenberg CGM. Dysregulated neutrophil-endothelial interaction in antineutrophil cytoplasmic autoantibody (ANCA)-associated vasculitides: Implications for pathogenesis and disease intervention. Autoimmun Rev. Elsevier B.V.; 2011;10: 536–543. doi:10.1016/j.autrev.2011.04.004

30.	Ludemann J, Utecht B, Gross WL. Anti-neutrophil cytoplasm antibodies in Wegener’s granulomatosis recognize an elastinolytic enzyme. J Exp Med. 1990;171: 357–362. doi:10.1084/jem.171.1.357

31.	Seibold F, Weber P, Klein R, Berg PA, Wiedmann KH. Clinical significance of antibodies against neutrophils in patients with inflammatory bowel disease and primary sclerosing cholangitis. Gut. 1992;33: 657–662. doi:10.1136/gut.33.5.657

32.	Gross WL, Hauschild S, Mistry N. The clinical relevance of ANCA in vasculitis. Clin Exp Immunol Suppl. 1993;93: 7–11. doi:10.1111/j.1365-2249.1993.tb06215.x

33.	Peen E, Almer S, Bodemar G, Ryden BO, Sjolin C, Tejle K, et al. Anti-lactoferrin antibodies and other types of ANCA in ulcerative colitis, primary sclerosing cholangitis, and Crohn’s disease. Gut. 1993;34: 56–62. doi:10.1136/gut.34.1.56

34.	Rivera-Correa J, Rodriguez A. Divergent Roles of Antiself Antibodies during Infection. Trends Immunol. 2018;39: 515–522. doi:10.1016/j.it.2018.04.003

35.	Cheng MP, Butler-Laporte G, Parkes LO, Bold TD, Fritzler MJ, Behr MA. Prevalence of Auto-antibodies in Pulmonary Tuberculosis. Open Forum Infect Dis. 2019;6: 1–5. doi:10.1093/ofid/ofz114

36.	Jansen TLTA, van Houte D, de Vries T, Wolthuis A. ANCA seropositivity in HIV: A serological pitfall. Neth J Med. 2005;63: 270–274.

37.	Worodria W, Menten J, Massinga-Loembe M, Mazakpwe D, Bagenda D, Koole O, et al. Clinical spectrum, risk factors and outcome of immune reconstitution inflammatory syndrome in patients with tuberculosis-HIV coinfection. Antivir Ther. 2012; doi:10.3851/IMP2108

38.	Worodria W, Massinga-Loembe M, Mayanja-Kizza H, Namaganda J, Kambugu A, Manabe YC, et al. Antiretroviral treatment-associated tuberculosis in a prospective cohort of HIV-infected patients starting ART. Clin Dev Immunol. 2011;2011: 758350. doi:10.1155/2011/758350

39.	(WHO) WHO. Improving the diagnosis and treatment of smear-negative pulmonary and extrapulmonary tuberculosis among adults and adolescents. Recommendations for HIV-prevalent and resource-contstrained settings. [Internet]. 2012. p. Available: http://libdoc.who.int/hq/2012/WHO_HTM_TB_2012.2_eng.pdf

40.	Meya D, Okurut S, Zziwa G, Cose S, Bohjanen P, Mayanja-Kizza H, et al. Monocyte Phenotype and IFN-γ-Inducible Cytokine Responses Are Associated with Cryptococcal Immune Reconstitution Inflammatory Syndrome. J Fungi. 2017;3: 28. doi:10.3390/jof3020028

41.	Garratt LW, Sutanto EN, Ling KM, Looi K, Iosifidis T, Martinovich KM, et al. Matrix metalloproteinase activation by free neutrophil elastase contributes to bronchiectasis progression in early cystic fibrosis. Eur Respir J. 2015;46: 384–394. doi:10.1183/09031936.00212114

42.	Goovaerts O, Jennes W, Massinga-Loembé M, Ondoa P, Ceulemans A, Vereecken C, et al. Lower pre-treatment T cell activation in early-and late-onset tuberculosis-associated immune reconstitution inflammatory syndrome. PLoS One. 2015;10: 1–15. doi:10.1371/journal.pone.0133924

43.	Goovaerts O, Jennes W, Massinga-Loembe M, Ceulemans A, Worodria W, Mayanja-Kizza H, et al. Antigen-Specific Interferon-Gamma Responses and Innate Cytokine Balance in TB-IRIS. PLoS.One. 2014. pp. e113101-.
